# Genome-guided and mass spectrometry investigation of natural products produced by a potential new actinobacterial strain isolated from a mangrove ecosystem in Futian, Shenzhen, China

**DOI:** 10.1038/s41598-018-37475-w

**Published:** 2019-01-29

**Authors:** Dini Hu, Cheng Gao, Chenghang Sun, Tao Jin, Guangyi Fan, Kai Meng Mok, Simon Ming-Yuen Lee

**Affiliations:** 1 Faculty of Science and Technology, Department of Civil and Environmental Engineering, University of Macau, Macao, China; 2 State Key Laboratory of Quality Research in Chinese Medicine and institute of Chinese Medical Sciences, University of Macau, Macao, China; 3 Institute of Medicinal Biotechnology, Chinese Academy of Medical Science & Peking Union Medical College, Tiantanxili No 1, Beijing, 100050 P. R. China; 4 Beijing Genome Institute–Shenzhen, Shenzhen, 518083 China; 5 Present Address: Institute of Chinese Medical Sciences, University of Macau, Avenida da Universidade, Room No N22-7003. N22 Building, Taipa, Macao China

## Abstract

Actinobacteria, a group of gram-positive bacteria, can produce plenty of valuable bioactive secondary metabolites, especially antibiotics. Hence, in order to search for new actinobacteria, actinobacterial isolates were obtained from rhizosphere soil collected from the Futian mangrove ecosystem in Shenzhen, China. According to 16S rRNA sequences, 14 actinobacterial strains of the genus *Streptomyces, Rhodococcus, Microbacterium*, *Micromonospora, Actinoplanes* and *Mycobacterium* were isolated and identified. Among these, strain *Mycobacterium* sp.13 was described as a potential new species belonging to the genus *Mycobacterium* within the class of actinobacteria according to the genomic analysis. The genome-based 16S rRNA sequences had 98.48% sequence similarity with *Mycobacterium moriokaense* DSM 44221^T^. Meanwhile, the genome sequences of *Mycobacterium* sp.13 showed an average nucleotide identity (ANI) with the *Mycobacterium mageritense* DSM 44476*, Mycobacterium smegmatis* MKD8 and *Mycobacterium goodii* strain X7B of only 74.79%, 76.12% and 76.42%, respectively. Furthermore, genome-mining results showed that *Mycobacterium* sp.13 contained 105 gene clusters encoding to the secondary metabolite biosynthesis, where many kinds of terpene, bacteriocin, T1pks, Nrps, saccharide, fatty acid, butyrolactone, ectoine and resorcinol were included. Finally, through LC-MS and HR-MS, analyzing the small molecules from ethyl acetate extract of this strain, asukamycin C and apramycin were for the first time found present to be in *Mycobacterium moriokaense* strain. Our study provides evidence in support of the potential new *Mycobacterium* sp.13 isolated from the mangrove environment as a possible novel source of natural products.

## Introduction

Natural products, including drugs, are clearly considered to occupy larger and distinct chemical spaces compared to combinatorial chemicals^1^. Natural products are derived from microorganisms, plants or animals^2,3^. In all known small molecule natural products producers, the microbial community represent a major source of biologically active secondary metabolites^4^. As we know, many important natural products isolated from bacteria that becoming potential leads for the human diseases treatment have been identified^5^. To date, about 20,000 secondary metabolites produced from microorganisms have been discovered^6^. Moreover, recent reports of new chemical entities and first-in-class drug candidates were frequently derived from actinobacterial strains^7,8^. As we know, *Streptomyces* is by far the most prolific genus, which produced about 80% of all described antibiotics^9^. However, the rare actinobacterial genus of *Mycobacterium* are little known to produce secondary metabolites^10^. Several mycobacteria, namely *Mycobacterium tuberculosis* and *Mycoabcterium smegmatis*, are only known to produce some sulfated compounds^11^. It is therefore, deducing the secondary metabolites producing ability by rare actinobacterial genus will require a substantial effort.

Increasing attention has already been placed on new or extreme environments for the exploration of novel natural compounds, such as marine^12^, desert^13^ and mangrove environments^14^. Mangroves are an ecosystem with high moisture, high salinity and tolerance to oxygen^15^. Many new types of microorganisms have been discovered from mangrove ecosystems, which have always been a natural source of secondary metabolites with biologically active. For example, A novel actinobacterial strain was isolated by Ruan *et al*. (2015) from mangrove soil in Thailand belonging to the genus *Streptomyces*^16^. A novel *Jiangella* strain was obtained by Suksaard *et al*. (2015) from a mangrove soil in 2015^17^. As we know, microbial community are valuable and rich sources of structurally peerless bioactive natural products^18^. In recent years, studies have obtained compounds from mangrove actinobacterial strains with a unique structure and potential medicinal applications. Such as, a novel analogue of 1-isoquinolone, described as marinamide (A), and its methyl ester (B), were isolated from two endophytic fungi^19^. More recently, two novel indolocarbazoles isolated from a mangrove-derived *Streptomyces* showed unprecedented structure of cyclic N-glycosidic bond between 1,3-carbon atoms within the glycosyl moiety and two indole nitrogen atoms in the indolocarbazole with enhanced anticancer activity^20^. Therefore, the mangrove ecosystem can be regarded as a new and diverse source of natural products that remains untapped.

The recent surge in genome-wide sequencing projects has once again yielded insight into the production of secondary metabolites, where the genetic data showed that the metabolic potential of these natural resources was seriously underestimated^21^. Thus, a promising method for drug discovery is based on combining genome mining analysis in genomic sequences to reveal the silent pathway for natural products^22^. The most widely studied model strain of *Streptomyces coelicolor* had been shown to contain more than 20 gene clusters involved with potential secondary metabolites genes in the genome^23^. With the intensification of genome sequencing and metabolite analysis efforts, the metabolic function of other actinobacterial taxa is gradually being explored. Recent progress in several rare actinobacterial genus had suggested that it had the same potential of secondary metabolites production as *Streptomyces*^24^. The complete genome of a marine actinobacterial species*, Salinispora tropica*, was released during 2007, which identified 15 secondary metabolite gene clusters, including the Salinosporamide A gene cluster, a potent anticancer agent^25^. McyLeod *et al*. (2016) found the genome of *Rhodococcus* sp RHA1 contained 24 non ribosomal peptide synthase genes and 7 polyketide synthase genes, in which, 6 genes exceed 25k bp, provided evidence that this strain harbored an extensive pathway of secondary metabolism^26^. All of these findings showed that the biosynthetic capability of natural products from microorganisms had been greatly underestimated by traditional methods of bioassay-guided natural product discovery^27^. Thus, a better understanding of the whole bacterial genome is the key to successfully identifying new natural products.

Accordingly, the aims of this research were (1) to obtain novel actinobacterial isolates from mangrove ecosystem in Futian National Reserve, Shenzhen by a plate culture method, and to characterize the isolates based on the 16S rRNA gene sequences; (2) to identify the biosynthetic gene cluster involved with antibiotic production by whole-genome sequencing; and (3) to determine small molecule metabolites in fermentation products of the isolated strain by LC-MS and HR-MS. The results of this study should provide reference data informing the discovery of major antibiotics producing strain for further research, which can also bring benefit to the pharmaceuticals industry.

## Materials and Methods

### Materials

All chemical reagents were purchased from Sigma Aldrich (USA). TIANamp Bacteria DNA Kit was purchased from TIANGEN Biotech (Beijing) Co., Ltd.

Isolation medias were used in this study including ISP media 2 (yeast extract, 4.0 g; dextrose, 4.0 g; malt extract, 10.0 g; agar, 20.0 g; distilled water 1 L, pH 7.2)^28^, ISP media 4 (starch, 10.0 g; NaCl, 1.0 g; K_2_PHO_4_, 1.0 g; (NH_4_)_2_SO_4_, 2.0 g; FeSO_4_.7H_2_O, 0.001 g; CaCO_3_, 2.0 g; MnCl_2_.4H_2_O, 0.001 g; ZnSO_4_.7H_2_O, 0.001 g; agar, 20.0 g; distilled water 1 L, pH 7.2)^29^, Gauze No. 1 (soluble starch, 20.0 g; ferrous sulfate, 0.01 g; sodium chloride, 0.5 g; potassium nitrate, 1.0 g; magnesium sulfate, 0.5 g; dipotassium hydrogen phosphate, 0.5 g; agar, 15.0 g; distilled water 1 L, pH 7.2)^30^, nutrient agar (peptone, 10.0 g; sodium chloride, 5.0 g; beef extract, 3.0 g; agar, 15.0 g; distilled water 1 L, pH 7.2)^31^, Czapek’s medium (sodium nitrate, 3.0 g; magnesium sulfate, 0.5 g; dipotassium hydrogen phosphate, 1.0 g; potassium chloride, 0.5 g; sucrose, 30.0 g; ferrous sulfate, 0.01 g; agar, 15.0 g; distilled water 1 L, pH 7.2)^32^ and halothiobacillus HL2 medium (glucose, 10.0 g; tryptone, 3.0 g; peptone, 5.0 g; NaCl, 5.0 g; agar, 20.0 g; distilled water 1 L, pH 7.2)^33^.

### Environmental sampling

The Futian National Nature Reserve, is situated at 22°32′ N and 114°05′ E and covers an area of about 369 ha, Shenzhen, the People’s Republic of China, Vegetation types include semi-mangroves, mangroves and seashore plants, with *Kandelia candel* and *Aegiceras corniculatum* being dominant in the Nature Reserve^34^. Samples were collected from rhizosphere soil, related to *Aegiceras corniculatum* and *Kandelia candel*, in August 2017. Collected soil samples were immediately placed in a sterile plastic bags and then transported them to the laboratory.

### Selective isolation procedures and medium

Air dried soil samples were firstly sieved to exclude organic matter particles and large mineral, then grounded in a mortar and pestle. Selective soil samples pretreatment method including dry heat (120 °C, 60 min) and phenol (1.5%, 30 min at 30 °C)^35^. The pretreated soil samples were serially diluted with steriziled water down to 10^–4^. Then, 100 μL amounts of the 10^−4^ suspensions were spread onto selected isolation medium. The culture-plate procedure was designed to isolate pure cultures of bacteria and conducted according to previous publication by Hu *et al*.^36^. Briefly, suspensions of each sample were diluted and spreaded onto six different kinds of isolation medium: ISP media 2, ISP media 4, Gauze No. 1, nutrient agar, Czapek’s medium and halothiobacillus HL2 medium. All isolation media were cultured and stored for future use.

### Extraction of DNA and PCR amplification

DNA was extracted directly from single colony to identify the species. The procedures of DNA extraction and PCR amplification were followed previous publication by Hu *et al*.^36^. DNA was extracted from the each single colony and used as the DNA template to do the PCR amplification^37^. The detailed PCR reaction for 16S rRNA amplification using the universal bacteria primer pair 27F (5′-AGAGTTTGATCCTGGCTCAG-3′) and 1492R (5′-TACGGCTACCTTGTTACGACTT-3′)^38^. The final products of PCR amplification were subsequently assessed in agarose gel by gel electrophoresis. Sanger sequencing platform was applied to sequence the products. The 16S rRNA sequences were compared with EzBioCloud database to determine the species (https://www.ezbiocloud.net/).

### Genome sequencing, assembly and annotation

The procedures of genomic DNA extraction, whole-genome sequencing and subsequently analysis were performed according to previous publication by Hu *et al*.^36^. Firstly, genomic DNA was extracted to construct the genomic DNA library, which was sequenced on the Illumina NovaSeq HiSeq. 4000. Then, genome assembly, annotation and gene prediction were performed by SOAPdenovo, idba and gmhmmp, respectively. Transfer RNAs (tRNAs) and ribosomal RNAs (rRNAs) were predicted by tRNAscan-SE and rnammer. All the protein-encoding genes were annotated by searching against the non-redundant protein sequence (NR) database of the National Center for Biotechnology Information (NCBI) using BLAST. Finally, the database of Kyoto Encyclopedia of Genes and Genomes (KEGG) was provided to assign the functional categories of the query sequences.

### Phylogenetic analysis and genome mining

The evolutionary relationships among different actinobacterial species were analyzed by Mega 7.0. 16S rRNA sequences of *Mycobacterium* sp.13 was retrieved from the genomic annotation results. In our study, we selected eight relevant mycobacteria species to construct the phylogenetic tree, whose sequences were downloaded from the NCBI database. An additional strain, *Nocardia abscessus* strain IMMIB D-1592 (Biosample accession: NR 025059.1) was used as the phylogenetic root^39^.

These species are *Mycobacterium moriokaense* strain CIP 105393 (Biosample accession: NR115331.1), *Mycobacterium goodii* strain ATCC 700504 (Biosample accession: AY457079.1), *Mycobacterium smegmatis* strain ATCC 19420 (Biosample accession: AY457078.1), *Mycobacterium mageritense* strain DSM 44476 (Biosample accession: AY457076.1), *Mycobacterium avium* strain JCM 15429 (Biosample accession: LC020093.1), *Mycobacterium ulcerans* strain ATCC 19423 (Biosample accession: NR113138.1), *Mycobacterium bovis* strain GTC 602 (Biosample accession: AB292583.1) and *Mycobacterium tuberculosis* strain UKR100 (Biosample accession: MG995565.1).

The online software of anti-SMASH was applied to mine the secondary metabolite biosynthesis gene clusters and predict the small molecule compounds production (https://antismash.secondarymetabolites.org/#!/about), which followed previous publication by Hu *et al*.^36^.

### Preparation of crude extract and LC-MS analysis

The procedures of crude extract preparation and subsequent MS analysis according to the previous work by Hu *et al*.^36^. Firstly, the purified culture was processed the fermentation culture following by the extraction with ethyl acetate. Then, MS/MS analysis was performed on a 4000 Q TRAP LC/MS/MS system. Furthermore, a high accurate mass spectrometric analysis was performed on a LTQ-Orbitrap mass spectrometer XL MS equipped with an ESI mode and operated in the positive ion. The mass accuracy of the instrument lower than 3 ppm. Full scan data was acquired from 500 to 600 m/z under the same condition.

### Nucleotide sequence accession numbers

The sequences of *Mycobacterium* sp.13 generated in this study have been deposited with the GenBank database under the accession number QQBJ00000000 for bacterial whole genome genes.

## Results

### Diversity of actinobacterial strains by 16S rRNA analysis

In total, 39 bacterial isolates were obtained from soil samples, 14 of which were related to the filamentous bacteria belonging to the class of actinobacteria (Supplementary Table S1 and Table 1). Isolates were identified by PCR and sequencing of 16S rRNA gene sequences, it turned out that they were classified into six genus in the class of actinobacteria, composing of *Micromonospora, Streptomyces, Mycobacterium, Microbacterium, Rhodococcus* and *Actinoallomurus*. Actinobacterial species were mainly isolated from five types of isolation media, e.i. ISP2, Nutrient agar, ISP4, HL2 and Gauze No. 1. Due to low 16S sequence identity with the nearest type strain (97.00%), one actinobacterial strain of *Mycobacterium* sp.13 was selected for further genomic analysis.

**Table 1:** Actinobacterial community isolated from the soil samples based on the 16S rRNA sequences.

Sample Type	Tree	Plates	No.	Top-hit taxon at species level	Similarity based on 16S (%)	Length (bp)
Rhizophere soil	*Kandelia candel*	ISP2	1	*Rhodococcus equi* NBRC 101255^T^	98.90	1095
		HL2	2	*Rhodococcus equi* NBRC 101255^T^	99.21	1015
		ISP4	3	*Microbacterium paraoxydans* NBRC 103076^T^	98.71	850
		HL2	4	*Actinoallomurus* acacia GMKU 931^T^	98.74	1112
		Gause No. 1	5	*Micromonospora aurantiaca* ATCC 27029^T^	99.76	850
		Nutrient agar	6	*Micromonospora aurantiaca* ATCC 27029^T^	99.76	850
		HL2	7	*Actinoallomurus acaciae* GMKU 931^T^	98.74	1112
	*Aegiceras corniculatum*	ISP2	8	*Streptomyces libani subsp. Libani* NBRC 13452^T^	99.09	1121
		HL2	9	*Streptomyces glauciniger* CGMCC 4.1858^T^	99.34	1069
		HL2	10	*Rhodococcus globerulus* NBRC 14531^T^	99.19	1110
		ISP4	11	*Micromonospora equina* Y22^T^	99.15	850
		Gause No. 1	12	*Mycobacterium conceptionense* CCUG 50187^T^	98.82	930
		ISP4	13	*Mycobacterium moriokaense* CIP 105393^T^	97.00	1041
		HL2	14	*Rhodococcus maanshanensis* DSM 44675^T^	99.35	1078

One of the actinobacteria strain (*Mycobacterium* sp.13) exhibited low 16S gene similarity (97.00%) and was further subjected to the whole genome sequencing.

### Genome mining and annotation

One of the isolated strains, *Mycobacterium* sp.13, the genome-based 16S rRNA gene isolated from strain, had 98.48% similarity to the sequence of *Mycobacterium moriokaense* DSM 44221^T^. Whole-genome sequencing of *Mycobacterium* sp.13 generated a total of 12,385,360 sequence reads, yielding approximately 268 scaffolds (Table 2). SOAPdenovo was used to do the *de novo* assembly, resulted in a total of 7.2 Mb (66.95% G+C content) distributed within one main scaffold having an average length of 26,880 bp. The assembled genome sequences were compared with the KEGG and NR databases to process the procedure of annotation. Within the *Mycobacterium* sp.13, a total of 7,144 protein-encoding genes were contained in the genome, 4 rRNA and 50 tRNA were predicted, the coding density was about 92.07% and the average CDS length was 928 bp. Strain *Mycobacterium* sp.13 also presented with low sequence similarity to other *Mycobacterium* strains, but these strains do not have genomic data within the NCBI database except for *Mycobacterium mageritense* DSM 44476*, Mycobacterium smegmatis* MKD8 and *Mycobacterium goodii* strain X7B. Therefore, these three strains were selected to calculate the average nucleotide identity (ANI), the value of which with the *Mycobacterium mageritense* DSM 44476, *Mycobacterium smegmatis* MKD8 and *Mycobacterium goodii* strain X7B was only 74.79%, 76.12% and 76.42%, respectively. The ANI result indicated that the isolate was a potential novel strain within the genus *Mycobacterium*, based on the genomic data.

**Table 2:** General features of the genomes of isolated *Mycobacterium* sp.13.

Sample	Mycobacterium sp.13
Length (bp)	7203886
N50 length (bp)	254710
N90 length (bp)	65463
Average length (bp)	26880.17
Coding density (%)	92.07%
Average CDS length (bp)	928.462206
No. of protein-coding genes	7144
No. of tRNA genes	50
No. of reads	12385360
No. of scaffolds	268
No. of rRNA	4
GC content	66.95%

### Phylogenetic analysis

**Figure 1:**
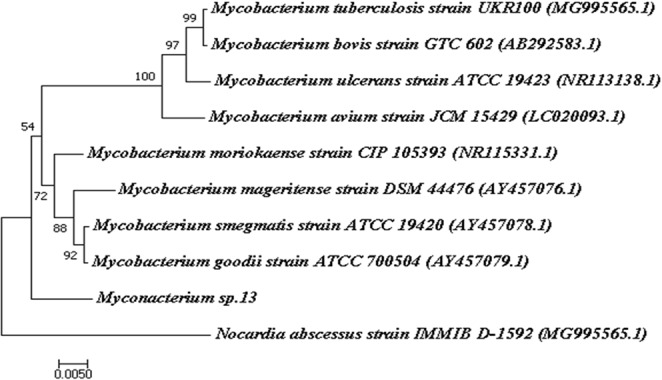
Comparison of eight 16S rRNA sequences from the genus of *Mycobacterium* with other orthologous sequences. Complete 16S rRNA sequence of the *Mycobacterium* sp.13 was extracted from the genome data. The tree is rooted with *Nocardia abscessus* strain IMMIBD-1592. The neighbor-joining method was used to construct the phylogenetic tree. The number of bootstrap replications was set to 1000.

A phylogenetic analysis was performed to assess the evolutionary relationship among different bacterial strains. In the tree, eight 16S sequences from the genus of *Mycobacterium* were compared. Phylogenetic analysis of *Mycobacterium* sp.13 based on 16S rRNA sequences showed that this potential novel species occupied a unique position at the genus level (Fig. 1). It was on a single branch, which was relatively close to *Mycobacterium goodii* strain ATCC 700504 and *Mycobacterium smegmatis* strain ATCC 19420. This suggested that *Mycobacterium* sp.13 had a close genetic relationship with them. The isolated *Mycobacterium* sp.13 was also close to *Mycobacterium mageritense* strain DSM 44476 and *Mycobacterium moriokaense* strain CIP 105393, but was relatively distant from *Mycobacterium bovis* strain GTC 602, *Mycobacterium avium* strain JCM 15429, *Mycobacterium ulcerans* strain ATCC 19423 and *Mycobacterium tuberculosis* strain UKR100.

### Detection of gene clusters involved in antibiotics and secondary metabolites

A total of 105 biosynthetic gene clusters related to the secondary metabolite production were identified in the genome of *Mycobacterium* sp.13 (Supplementary Table S2), which were predicted for T1pks, terpene, Nrps, fatty acid, bacteriocin, ectoine, butyrolactone, saccharide, resorcinol and other products, in which 6 gene clusters showed similarity exceeding 20%. The analysis showed that at least 10 kinds of PKS and NRPS gene clusters can be observed in the *Mycobacterium* sp.13 genome, 3 of which had no similarity with any known gene cluster and the core structures of these clusters were identified (Table 3). In addition, seven of the biosynthetic gene clusters were encoded to antibiotics compounds, namely sch47554/sch47555, azinomycin B, apramycin, maklamicin, u-68204, nosiheptode and caprazamycin (Table 4).

**Table 3:** Overview of 10 secondary metabolites of biosynthetic PKS/NRPS gene clusters of *Mycobacterium* sp.13, detected by anti-SMASH.

Type	Region	Most similar known cluster	Homology (%)	Reference strain (Gene Bank ID)	Core structure	Reference
T1pks-Nrps	305280–363883	Glycopeptidolipid	26	*Mycobacterium smegmatis* (AY439015)		^69^
T1pks	1–39994	Asukamycin	4	*Streptomyces nodosus* ATCC 29757 (GQ926890)		^70^
Nrps	124058–201141					
T1pks-Butyrolactone	308529–360681					
T1pks-Resorcinol	19660–64983	Sch47554/Sch47555	7	*Streptomyces* sp. SCC 2136 (AJ628018)		^71^
Nrps	227646–252490	Glycopeptidolipid	10	*Mycobacterium smegmatis* (AY439015)		^69^
T1pks-saccharide	56964–142329					
T1pks	1–24838	Maklamicin	4	*Micromonospora* sp. GMKU326 (LC021382)		^58^
Saccharide-Nrps	200195–248739	Glycopeptidolipid	94	*Mycobacterium abscessus* strain CIP104536^T^ (AM231618)		^69^
Nrps	1–7827	Glycopeptidolipid	7	*Mycobacterium avium* strain 2151 (AF143772)

**Table 4:** Overview of seven secondary metabolites of biosynthetic gene clusters involved in the production of antibiotic compounds of *Mycobacterium* sp.13, detected by anti-SMASH.

Type	Region	Most similar known cluster	Homology (%)	Antibiotic types	Reference strain (Gene Bank ID)	Structure	References
T1pks-Resorcinol	19660–64983	Sch47554/Sch47555	7	Antifungal antibiotics	*Streptomyces* sp. SCC 2136 (AJ628018)		^71^
Putative	173613–64983	Azinomycin B	4	Macrolide antibiotics	*Streptomyces sahachiroi* (EU240558)		^72^
Putative	433402–459003	Apramycin	6	Aminoglycoside antibiotic	*Streptoalloteichus hindustanus* DSM 44523^T^ (AJ875019)		^73^
T1pks	1–24838	Maklamicin	4	Spirotetronate-class antibiotic	*Micromonospora* sp. GMKU326 (LC021382)		^58^
Fatty acid	1–19122	U-68204	14	Thiolactone-containing antibiotic	*Streptomyces* sp. MG11 (LN879416)		^59^
Putative	330168–358312	Nosiheptide	15	Thiopeptide antibiotic	*Streptomyces actuosus* (FJ438820)		^74^
Fatty acid	33337–63071	Caprazamycin	28	Anti-tuberculosis antibiotic	*Streptomyces* sp MK730–62F2 (FJ490409)		^75^

The antibiotic of the maklamicin biosynthesis gene cluster of *Mycobacterium* sp.13, contained 20 ORFs, and three catalytic domains related to the polyketide synthase (Supplementary Fig. 1). In the genome of *Mycobacterium* sp.13, core secondary biosynthetic genes were related to AMP-dependent synthase and ligase, phosphopantetheine-binding domain-containing protein and thioesterase, additional biosynthetic genes were annotated to aminotransferase class V, GCN5-related N-acetyltransferase, pyruvate oxidase/decarboxylase and monooxygenase FAD-binding; some regulatory and transport-related genes can also be observed in the *Mycobacterium* sp.13 genome. In addition, maklamicin biosynthesis clusters of *Mycobacterium* sp.13 had 4% homology to the existing cluster of *Micromonospora* sp GMKU326. This may play an important role in the synthesis of the maklamicin-derived primer unit, which meant that these clusters may be derived from donor microorganisms via horizontal gene transfer. Another PKS-type biosynthetic gene cluster contained 42 ORFs and four domains related to antibiotic sch47554/sch47555 production (Supplementary Fig. 2). In sch47554/sch47555-like gene cluster, core and additional biosynthetic genes were encoded to rhodanese domain-containing protein, AMP-dependent synthetase and ligase, MMPL domain-containing transport protein, beta-ketoacyl synthase, phosphopantetheine-binding domain-containing protein, acyl carrier protein, 3-oxoacyl, o-methyltransferase, monooxygenase FAD-binding, phosphoglycerate mutase, isochorismate synthase, argininosuccinate lyase/adenylosuccinate lyase and alpha/beta hydrolase fold protein. Sch47554/sch47555 biosynthetic gene cluster was also constituted of considerable proteins with unknown function. Therefore, *Mycobacterium* sp.13 had great potential for producing an analogue of sch47554/sch47555.

In addition to the analysis of PKS type cluster, the fatty acid type gene clusters responsible for antibiotic synthase were found in the genome of *Mycobacterium* sp.13. The first fatty acid gene cluster related to caprazamycin-like compound production was consisted of 30 ORFs, 3 of which encoding key biosynthetic genes including 3-oxoacyl and NAD-dependent epimerase/dehydratase (Supplementary Fig. 3). This cluster of *Mycobacterium* sp.13 had different biosynthetic enzymes compared with the most-similar known cluster in the genome of *Streptomyces* sp MK730-62F2, which indicated that an unknown compound or caprazamycin analog might can be discovered from the new strain isolated from mangrove environment. Another fatty acid pathway was annotated to synthesis of U-68204 (Supplementary Fig. 4). It is worth noting that numerous genes in the U-68204-like gene cluster cannot be annotated. The core biosynthetic enzymes were related to malonyl CoA-acyl carrier protein transacylase, beta-ketoacyl synthase and putative acyl carrier protein.

Besides the fatty acid and PKS type gene clusters, other putative gene clusters can be found in the genome of *Mycobacterium* sp.13, which were also responsible for antibiotic production including of nosiheptode, apramycin and azinomycin B. The nosiheptode biosynthesis gene cluster of *Mycobacterium* sp.13 contained 24 ORFs (Supplementary Fig. 5). However, this cluster of *Mycobacterium* sp.13 had more annotated functional known biosynthetic genes involved with the antibiotics production than the *Streptomyces actuosus* cluster. In the genome of *Mycobacterium* sp.13, core biosynthetic genes were related to the putative esterase and glycosyltransferase. Furthermore, biosynthetic gene clusters of apramycin and azinomycin B were discovered in *Mycobacterium* sp.13 (Supplementary Figs 6 and 7). In the azinomycin biosynthetic gene cluster, the core genes were related to the synthase of haloalkane dehalogenase, oxidoreductase and short-chain dehydrogenase/reductase SDR. No core biosynthetic genes were observed in the genome responsible for apramycin biosynthesis.

### Detection of secondary metabolites produced by *Mycobacterium* sp.13

**Figure 2:**
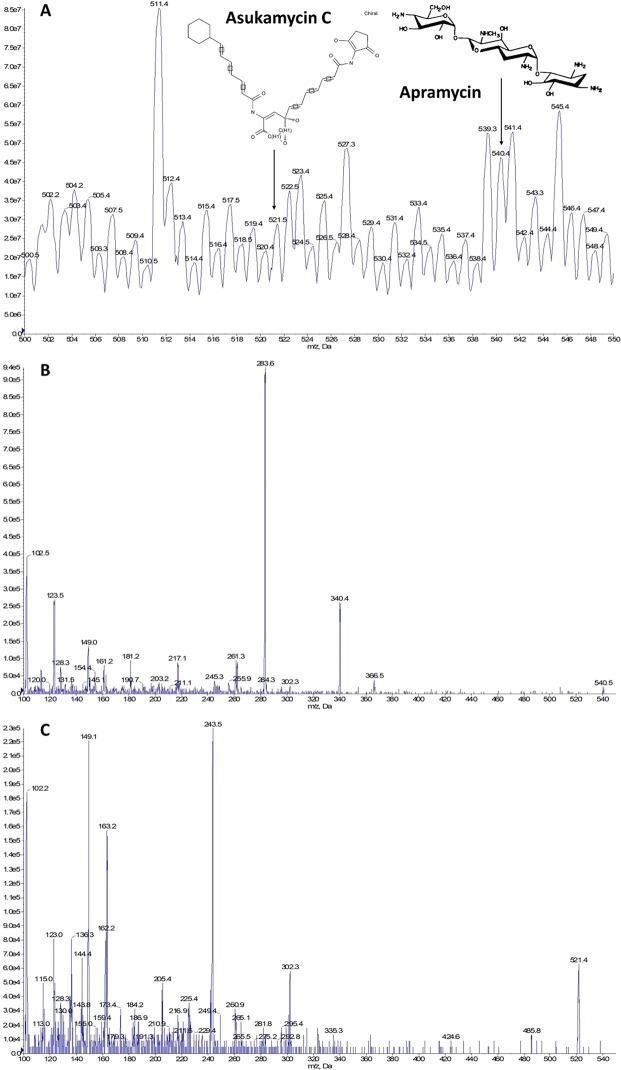
LC-MS analysis of ethyl acetate (EA) extract of asukamycin C and apramycin in the fermentation broth of *Mycobacterium* sp.13. The apramycin ([M + H]^+^ at m/z 540.4 (A), in-source fragment at m/z 217.1 (**B**)) and asukamycin C ([M + H]^+^ at m/z 521.5 (**A**), in-source fragment at m/z 163.2 (**C**)) were identified.

**Figure 3:**
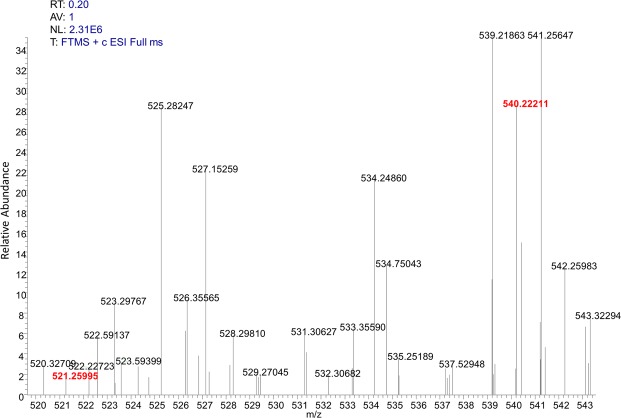
HR-MS analysis of ethyl acetate (EA) extract of asukamycin C and apramycin in the fermentation broth of *Mycobacterium* sp.13. The apramycin ([M + H]^+^ at m/z 540.22211) and asukamycin C ([M + H]^+^ at m/z 521.25995) were identified.

To test the results of genome-guided secondary metabolites prediction, a small-molecule profile was established. Mass spectral analysis were performed in positive mode. According to the mass-to-charge ratio of molecular ions, the metabolic substances in the fermentation broth were identified from the UPLC-MS profiles. After that, obtained profiles of secondary metabolites by mass spectrometry were compared with the genome mining data. As thus, two predicted secondary metabolites were detected in the fermentation product of *Mycobacterium* sp.13: asukamycin C (cluster 25, m/z = 521.5[M + H]^+^)^40^ and apramycin (cluster 39, m/z = 540.4 [M + H]^+^)^41^ (Fig. 2A). Corresponding fractions were also observed, in-source fragments at m/z 217.1 correspond to apramycin (Fig. 2B) and m/z 163.2 correspond to asukamycin C (Fig. 2C) were identified. Furthermore, the accurate identification of the compounds was conducted by HR-MS with high mass accuracy. The theoretical exact mass in positive mode was calculated by ChemDraw used as the reference in our study. The theoretical exact mass of MH^+^ (m/z) of asukamycin C and apramycin was 521.22878 (C_29_H_33_N_2_O_7_^+^) and 540.28809 (C_21_H_42_N_5_O_11_^+^), respectively. The measured mass using HR-MS was 521.25092 and 540.22180, which was shown in Fig. 3.

## Discussion

Currently, tremendous research interest is concentrated on the isolation and identification of new actinobacterial species from unique and extreme environmental surroundings, as the screening of these microorganisms has increased the prospect of exploring novel natural products that could be exploited as biotechnological resources^42,43^. Such as, four hundred and forty eight actinobacterial strains were isolated by Suksaard *et al*. (2017) from water and sediment samples of mangroves and assessed the plant growth promoting potential of them^44^. Ruttanasutja *et al*. (2015) designed different isolation method to isolate diverse actinobacterial strains, including five pretreatments process, three enrichment media and fifteen selective media^45^. For this purpose, a mangrove forest was selected as the sampling site for this study, which was located in the regions along estuaries with tropical and subtropical climate and proved to be the driving force of bacterial diversity^46^. In our study, the soil used in the experiment were collected from Futian mangrove ecosystem in Shenzhen, China, which located in a tidal swamp of tropical delta. The 16S rRNA sequences were amplified by universal primers and then sequenced via Sanger sequencing platform to identify the species^47^. The isolation results indicated that 39 bacterial isolates were obtained from rhizosphere soil samples, 14 isolates of which were identified as the actinobacterial strains belonging to six genera: *Streptomyces, Micromonospora, Mycobacterium, Microbacterium, Actinoallomurus* and *Rhodococcus*. Among them, one *Mycobacterium* sp.13 only presented 97.00% 16S sequence similarity with *Mycobacterium moriokaense*. The 16S rRNA gene can be used for species classification as it is a highly conserved gene; the application of the 98.65% threshold could strictly classify bacterial community into different species depending on the 16S rRNA gene copy analyzed^48^. Thus, *Mycobacterium* sp.13 was selected to perform the whole genome sequencing. The complete 16S rRNA sequences obtained from the genome had 98.48% sequence similarity with *Mycobacterium moriokaense* DSM 44221^T^. Therefore, this isolate could be regarded as a potential new actinobacterial strain and was subjected to further genome mining. The genome sequence of the potential new actinobacterial strain showed an ANI with the *Mycobacterium mageritense* DSM 44476, *Mycobacterium smegmatis* MKD8 and *Mycobacterium goodii* strain X7B of only 74.79%, 76.12% and 76.42%, respectively. Among the available indices of genome-relatedness, the ANI value between two bacterial genome sequences is one of the most robust measurements of genome correlations and has great potential as a compensation for the DNA–DNA hybridization (DDH) technique in the classification of bacteria and archaea^49^. The ANI threshold range for species delineation (95–96%) has been determined based on a comparative study between the DDH and ANI values^50^. On account of genome-based results, *Mycobacterium* sp.13 can be classified as a potential novel species within the genus of *Mycobacterium*.

Previous efforts in genomic analysis have proven that actinobacterial strain isolated from mangrove environment could have a greater gene clusters involved in multiple secondary metabolic pathway compared with normal strains^36^. This observation has led us to explore this potential novel species isolated from mangrove which should offer a higher probability of discovering novel compounds than previously screened strains. The results of genome mining showed that *Mycobacterium* sp.13 had the capacity to produce such diverse antibiotics compounds, such as sch47554/sch47555, azinomycin B, apramycin, maklamicin, u-68204, nosiheptode and caprazamycin. The identification of biosynthetic gene cluster in *Mycobacterium* sp.13 genome revealed that a total of 105 gene clusters were related to the secondary metabolites production, which was significantly higher than other actinobacterial model strain. To date, roughly more than 2,000 actinobacterial strains genomes have been completed and annotated; these have been deposited in the database of NCBI. Homology searching for genes encoding the known or putative gene clusters responsible for secondary metabolite production revealed that *Streptomyces coelicolor* A3(2) predicted a further 17 gene clusters related to secondary metabolism^23^; *Streptomyces avermitilis* MA-4680 harbored 37 secondary metabolic gene clusters^51^, and the genome of *Saccharopolyspora erythraea* NRRL2338 suggested at least 27 biosynthesis genes involved with a various of secondary metabolites of mostly unknown composition of chemical compounds^52^. As noted before, the best-known *Mycobacterium* species in previous reports are pathogenic; there have been almost no reports of extensive antibiotics being derived from it, even belonging to the actinobacterial taxon^53–55^. In our study, the *Mycobacterium* sp.13 genome had rich genetic potential for secondary metabolite production; at least seven different kinds of antibiotic compound were found by the biosynthetic gene cluster prediction. In addition, there were many putative gene clusters in the genome involving secondary metabolites, and many predicted secondary metabolites were previously found to be isolated from the non-*Mycobacterium* strain. For example, the gene organization of polyketide azinomycin B was quite similar to that in *Streptomyces sahachiroi*^56^. The other predicted antibiotics of sch47554/sch47555, apramycin, maklamicin, u-68204, nosiheptode and caprazamycin were commonly found in *Streptomyces* sp SCC 2136*, Streptoalloteichus hindustanus* DSM 44523^T^, *Micromonospora* sp GMKU326*, Streptomyces* sp MG11*, Streptomyces actuosus* and *Streptomyces* sp MK730-62F2, respectively^57–61^. These observations indicated that this new strain had great potential for biosynthesis of diverse secondary metabolites, likely via different synthetic pathways compared to other known *Mycobacterium* species. All these observations proved that a complex and diverse secondary metabolome existed within the *Mycobacterium* strain genome. Genome mining efforts in our study indicated that the capability of *Mycobacterium* to produce secondary metabolites had been underestimated.

With the availability of the genome sequences of *Mycobacterium* sp.13, we are now in a position to address the possible secondary metabolites. Based on this, analysis of fermentation product of the *Mycobacterium* sp.13 can led to identification of the secondary metabolites asukamycin C and apramycin, which will further validate the genome mining prediction results. Asukamycin was isolated from the fermentation culture broth of a *Streptomyces* designated as *Streptomyces nodosus subsp. Asukaensis* in 1976^62^, which was a member of a potential antitumor agent within the manumycin family of metabolites^63^. In addition, apramycin, an aminocyclitol antibiotic complex produced by *Streptomyces tenebrarius*, was first reported in 1967^64^. *In vitro* experiment proved that apramycin can act against multidrug-resistant *Pseudomonas aeruginosa* and *Acinetobacter baumannii*^65^. To our knowledge, this study represented a first record of asukamycin C and apramycin being produced from the *Mycobacterium moriokaense* strain. In fact, few secondary metabolites have been described as produced by strains belonging to the genus of *Mycobacterium*, because the cell wall of them is shown to be an effective permeability barrier to hydrophilic compounds^66^. On the other hand, the expression of secondary metabolites gene clusters is regulated by many different protein families, and led to a range of secondary metabolites with varying concentrations^67^. Thus, a step in this direction is to develop a method to identify the secondary metabolites that share common structural features^68^. Although our study does not provide the absolute information on identifying the complete structure of the metabolites, some of fragments of metabolites can be observed by MS/MS spectrum. Therefore, an integrative genome- and LC-MS-based metabolomics strategy could facilitate the screening of natural products from actinobacterial strain.

## Supplementary information

Dataset 1
