# Supplementary material for: Genome-guided and mass spectrometry investigation of natural products produced by a potential new actinobacterial strain isolated from a mangrove ecosystem in Futian, Shenzhen, China
Source: Sci Rep. 2019 Jan 29;9:823. doi: 10.1038/s41598-018-37475-w (PMC6351551; doi:10.1038/s41598-018-37475-w)
Supplement: Supplementary file 1 — Dataset 1 [file 41598_2018_37475_MOESM1_ESM.pdf]

Supplementary Information

Genome-guided and mass spectrometry investigation of natural products produced by a potential new actinobacterial strain isolated from a mangrove ecosystem in Futian, Shenzhen, China

Hu Dini[1]; Gao Cheng[2]; Sun Chenghang[3]; Jin Tao[4]; Fan Guangyi[4]; Mok Kai Meng[1] and Lee Simon Ming-Yuen[2]

1 Faculty of Science and Technology, Department of Civil and Environmental Engineering, University of Macau, Macao, China;

2 State Key Laboratory of Quality Research in Chinese Medicine and institute of Chinese Medical Sciences, University of Macau, Macao, China;

3 Institute of Medicinal Biotechnology, Chinese Academy of Medical Science & Peking Union Medical College, Tiantanxili No 1, Beijing 100050, P.R. China;

4 Beijing Genome Institute–Shenzhen, Shenzhen 518083, China.

These authors contributed equally to this work

Correspondence and requests for materials should be addressed to S.M.L (simonlee@umac.mo)

Supplementary Tables

Table S1. Isolated bacterial community from the soil samples based on the 16S rRNA sequences.

Sample Types	Tree	Plates	Top-hit taxon	Top-hit strain	Similarity based on 16s (%)	Length (bp)
Soil	*Kandelia candel*	ISP2	*Rhodococcus equi*	NBRC 101255^{T}	98.90	1095
Soil	*Kandelia candel*	HL2	*Rhodococcus equi*	NBRC 101255^{T}	99.21	1015
Soil	*Kandelia candel*	ISP4	*Microbacterium paraoxydans*	NBRC 103076^{T}	98.71	850
Soil	*Kandelia candel*	HL2	*Actinoallomurus acaciae*	GMKU 931^{T}	98.74	1112
Soil	*Kandelia candel*	ISP2	*Stenotrophomonas panacihumi*	JCM 16536^{T}	99.53	850
Soil	*Kandelia candel*	ISP4	*Bacillus cibi*	DSM 16189^{T}	97.30	1050
Soil	*Kandelia candel*	ISP4	*Bacillus vietnamensis*	15-1^{T}	98.62	1017
Soil	*Kandelia candel*	Gause No.1	*Micromonospora aurantiaca*	ATCC 27029^{T}	99.76	850
Soil	*Kandelia candel*	Nutrient agar	*Micromonospora aurantiaca*	ATCC 27029^{T}	99.76	850
Soil	*Kandelia candel*	HL2	*Actinoallomurus acaciae*	GMKU 931^{T}	98.74	1112
Soil	*Kandelia candel*	ISP4	*Bacillus aryabhattai*	B8W22^{T}	99.35	930
Soil	*Kandelia candel*	Czapek	*Paenibacillus nebraskensis*	JJ-59^{T}	98.81	930
Soil	*Kandelia candel*	HL2	*Bacillus aryabhattai*	B8W22^{T}	99.25	930

Soil	*Kandelia candel*	ISP4	*Bacillus vietnamensis*	15-1^T	97.83	881
Soil	*Kandelia candel*	ISP4	*Bacillus vietnamensis*	15-1^T	98.58	850
Soil	*Kandelia candel*	Gause No.1	*Paenibacillus alginolyticus*	DSM 5050^T	98.45	1109
Soil	*Kandelia candel*	ISP2	*Bacillus drentensis*	LMG 21831^T	100.00	1046
Soil	*Kandelia candel*	Czapek	*Bacillus drentensis*	LMG 21831^T	100.00	930
Soil	*Kandelia candel*	ISP4	*Adhaeribacter terreus*	DNG6^T	97.20	930
Soil	*Aegiceras corniculatum*	ISP2	*Streptomyces libani subsp. libani*	NBRC 13452^T	99.09	1121
Soil	*Aegiceras corniculatum*	HL2	*Streptomyces glauciniger*	CGMCC 4.1858^T	99.34	1069
Soil	*Aegiceras corniculatum*	HL2	*Rhodococcus globerulus*	NBRC 14531^T	99.19	1110
soil	*Aegiceras corniculatum*	ISP4	*Micromonospora equina*	Y22^T	99.15	850
Soil	*Aegiceras corniculatum*	Gause No.1	*Mycobacterium conceptionense*	CCUG 50187^T	98.82	930
Soil	*Aegiceras corniculatum*	ISP4	*Mycobacterium moriokaense*	CIP 105393^T	97.00	1041
Soil	*Aegiceras corniculatum*	HL2	*Rhodococcus maanshanensis*	DSM 44675^T	99.35	1078
Plant	*Aegiceras*	HL2	*Serratia nematodiphila*	DSM 21420^T	99.06	850

	corniculatum					
Plant	*Aegiceras corniculatum*	Nutrient agar	*Pseudomonas paralactis*	WS4992^{T}	99.65	850
Soil	*Aegiceras corniculatum*	Nutrient agar	*Bacillus pacificus*	EB422^{T}	99.18	850
Plant	*Aegiceras corniculatum*	ISP2	*Pseudomonas simiae*	OLi^{T}	99.63	850
Soil	*Aegiceras corniculatum*	ISP4	*Paenibacillus xylanexedens*	B22a^{T}	99.03	930
Soil	*Aegiceras corniculatum*	ISP4	*Paenibacillus nebraskensis*	JJ-59^{T}	98.35	850
Soil	*Aegiceras corniculatum*	ISP2	*Massilia aerilata*	5516S-11^{T}	99.76	850
Soil	*Aegiceras corniculatum*	ISP4	*Massilia arvi*	THG-RS2O^{T}	98.62	1026
Soil	*Aegiceras corniculatum*	ISP4	*Massilia aerilata*	5516S-11^{T}	98.62	1109
Soil	*Aegiceras corniculatum*	ISP4	*Bacillus vietnamensis*	15-1^{T}	98.53	1101
Soil	*Aegiceras corniculatum*	ISP4	*Bacillus vietnamensis*	15-1^{T}	98.90	1008
Soil	*Aegiceras*	ISP4	*Bacillus hwajinpoensis*	SW-72^{T}	98.14	913

	corniculatum					
Soil	*Aegiceras corniculatum*	ISP4	*Massilia aerilata*	5516S-11^{T}	99.78	929

Table S2. Overview of 105 secondary metabolites of biosynthetic gene clusters involved in the production of secondary metabolites of *Mycobacterium* sp.13, detected by anti-SMASH.

Cluster	Type	From	To	Most similar known cluster
Cluster 1	Putative	39394	49846	
Cluster 2	Putative	67672	86246	
Cluster 3	Putative	217855	248773	
Cluster 4	Putative	257445	293886	
Cluster 5	T1pks-Nrps	305280	363883	Glycopeptidolipid biosynthetic gene cluster (26% of genes show similarity)
Cluster 6	Putative	379975	391567	
Cluster 7	Putative	399485	407373	
Cluster 8	Putative	425304	438184	
Cluster 9	Fatty acid	1	19122	U-68204 biosynthetic gene cluster (14% of genes show similarity)
Cluster 10	Putative	22537	41268	

Cluster 11	Putative	217781	224843	K-252a biosynthetic gene cluster (8% of genes show similarity)
Cluster 12	Putative	246515	251955	
Cluster 13	Putative	287230	299920	Herboxidiene biosynthetic gene cluster (3% of genes show similarity)
Cluster 14	Putative	46371	64801	
Cluster 15	Putative	68410	101680	
Cluster 16	Fatty acid	107115	161017	Polyoxypeptin biosynthetic gene cluster (10% of genes show similarity)
Cluster 17	Putative	172942	192204	
Cluster 18	Putative	198197	209562	
Cluster 19	Putative	216774	232690	
Cluster 20	Putative	236839	256018	Meridamycin biosynthetic gene cluster (4% of genes show similarity)
Cluster 21	Putative	325438	335682	
Cluster 22	Putative	349085	381455	
Cluster 23	Putative	404398	410344	
Cluster 24	Putative	415521	419199	
Cluster 25	T1pks	1	39994	Asukamycin biosynthetic gene cluster (4% of genes show similarity)
Cluster 26	Putative	100915	109108	
Cluster 27	Putative	112840	139772	

Cluster 28	Putative	143411	169684	Esmeraldin biosynthetic gene cluster (8% of genes show similarity)
Cluster 29	Putative	295656	304740	
Cluster 30	Putative	355259	367549	
Cluster 31	Putative	371405	382998	
Cluster 32	Saccharide	14476	84084	
Cluster 33	Nrps	124058	201141	
Cluster 34	Putative	208951	224388	
Cluster 35	Bacteriocin	239802	250599	
Cluster 36	Putative	276430	290005	
Cluster 37	T1pks-Butyrolactone	308529	360681	
Cluster 38	Putative	361004	376556	
Cluster 39	Putative	433402	459003	Apramycin biosynthetic gene cluster (6% of genes show similarity)
Cluster 40	Fatty acid	27828	57082	
Cluster 41	Putative	64484	79079	
Cluster 42	Other	99921	150222	
Cluster 43	Putative	156713	171921	
Cluster 44	Putative	182329	191718	
Cluster 45	Putative	197498	221319	Thiotetroamide biosynthetic gene cluster (11% of genes show similarity)

Cluster 46	Putative	227400	237745	Arsenopolyketides biosynthetic gene cluster (8% of genes show similarity)
Cluster 47	Putative	246369	276738	
Cluster 48	Ectoine	13174	35327	Ectoine biosynthetic gene cluster (66% of genes show similarity)
Cluster 49	Putative	59535	68162	
Cluster 50	Fatty acid	155924	184664	
Cluster 51	Putative	211428	255052	Phosphonoglycans biosynthetic gene cluster (3% of genes show similarity)
Cluster 52	Putative	255843	270767	
Cluster 53	Saccharide	53640	77204	
Cluster 54	Putative	173613	195787	Azinomycin B biosynthetic gene cluster (4% of genes show similarity)
Cluster 55	T1pks-Resorcinol	19660	64983	Sch47554/Sch47555 biosynthetic gene cluster (7% of genes show similarity)
Cluster 56	Saccharide	111916	163252	
Cluster 57	Putative	174835	181850	
Cluster 58	Putative	203111	212631	
Cluster 59	Nrps	227646	252490	Glycopeptidolipid biosynthetic gene cluster (10% of genes show similarity)
Cluster 60	Terpene	1	14976	Isorenieratene biosynthetic gene cluster (71% of genes show similarity)

Cluster 61	T1pks-Cf_saccharide	56964	142329	
Cluster 62	Putative	158416	169340	
Cluster 63	Putative	42795	53643	Phosphonoglycans biosynthetic gene cluster (3% of genes show similarity)
Cluster 64	Putative	71294	96798	
Cluster 65	Putative	125763	140161	
Cluster 66	Putative	211311	223095	
Cluster 67	Putative	49821	65311	
Cluster 68	Putative	307167	325198	Mycolic acid biosynthetic gene cluster (100% of genes show similarity)
Cluster 69	Putative	330168	358312	Nosiheptide biosynthetic gene cluster (15% of genes show similarity)
Cluster 70	Putative	29273	40298	
Cluster 71	T1pks	1	24838	Maklamicin biosynthetic gene cluster (4% of genes show similarity)
Cluster 72	Putative	71951	79923	
Cluster 73	Fatty acid	123156	144190	
Cluster 74	Putative	2	23058	
Cluster 75	Putative	85169	102685	
Cluster 76	Saccharide	114790	151099	
Cluster 77	Putative	51024	57531	

Cluster 78	Putative	82124	99304	
Cluster 79	Putative	122692	138301	
Cluster 80	Putative	8757	15796	
Cluster 81	Fatty acid	33337	63071	Caprazamycin biosynthetic gene cluster (28% of genes show similarity)
Cluster 82	Putative	163575	184285	
Cluster 83	Saccharide-Nrps	200195	248739	Glycopeptidolipid biosynthetic gene cluster (94% of genes show similarity)
Cluster 84	Putative	76531	94223	
Cluster 85	Other	30828	74760	Phosphonoglycans biosynthetic gene cluster (3% of genes show similarity)
Cluster 86	Putative	99499	114234	
Cluster 87	Putative	69258	84563	
Cluster 88	Putative	52702	62049	
Cluster 89	Putative	10356	16403	
Cluster 90	Putative	89542	100338	
Cluster 91	Putative	3159	31340	
Cluster 92	Putative	35054	45961	
Cluster 93	Putative	93272	97140	
Cluster 94	Putative	85108	97668	
Cluster 95	Other	32260	74755	
Cluster 96	Putative	1346	5995	

Cluster 97	Putative	42243	58811	
Cluster 98	Putative	590	5094	
Cluster 99	Putative	7013	14333	
Cluster 100	Putative	25954	46597	
Cluster 101	Putative	30187	42542	
Cluster 102	Putative	5795	15419	
Cluster 103	Putative	15210	26047	
Cluster 104	Other	1	17459	
Cluster 105	Nrps	1	7827	Glycopeptidolipid biosynthetic gene cluster (7% of genes show similarity)

Supplementary Figures

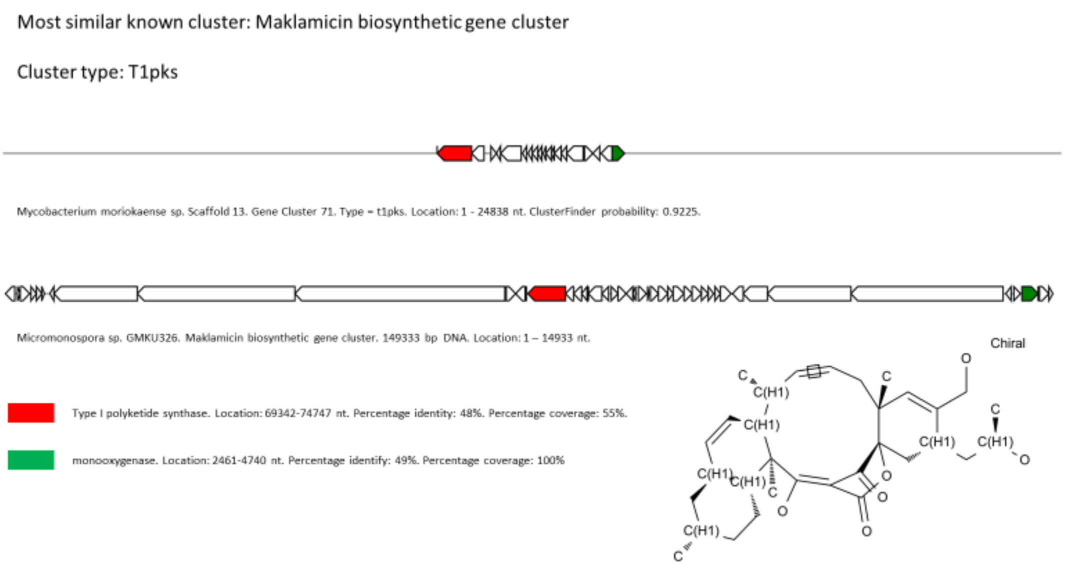

Figure S1. Proposed biosynthetic gene cluster of maklamicin-like compound in *Mycobacterium* sp.13. The most similar gene cluster from *Micromonospora* sp. GMKU326 is shown, with related genes drawn in the same color to highlight inter-cluster rearrangements. The detailed structure of the predicted compound is also displayed.

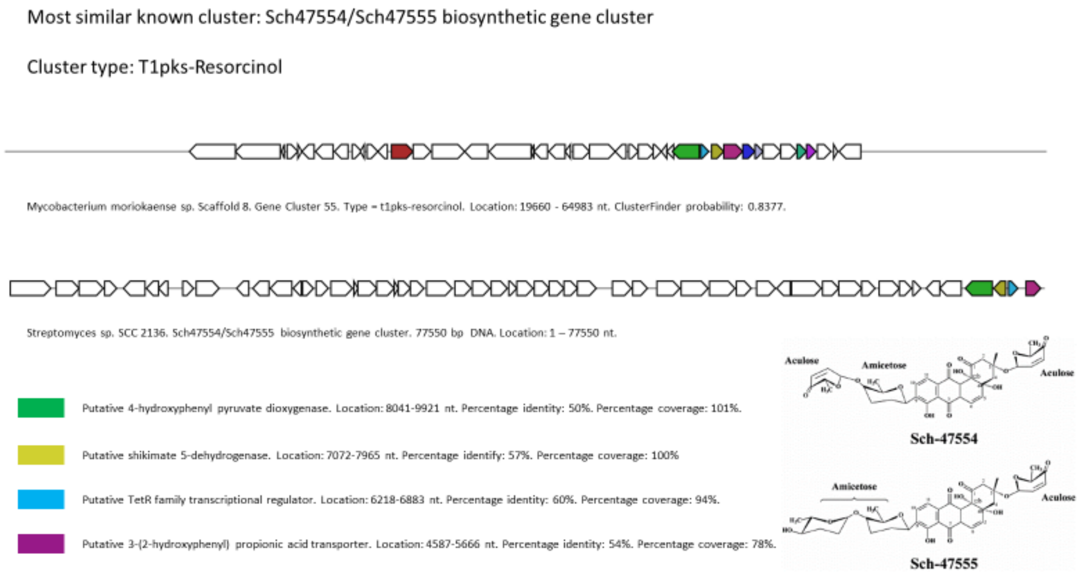

Figure S2. Proposed biosynthetic gene cluster of sch47554/sch47555-like compound in *Mycobacterium* sp.13. The most similar gene cluster from *Streptomyces* sp. SCC 2136 is shown, with related genes drawn in the same color to highlight inter-cluster rearrangements. The detailed structure of the predicted compound is also displayed.

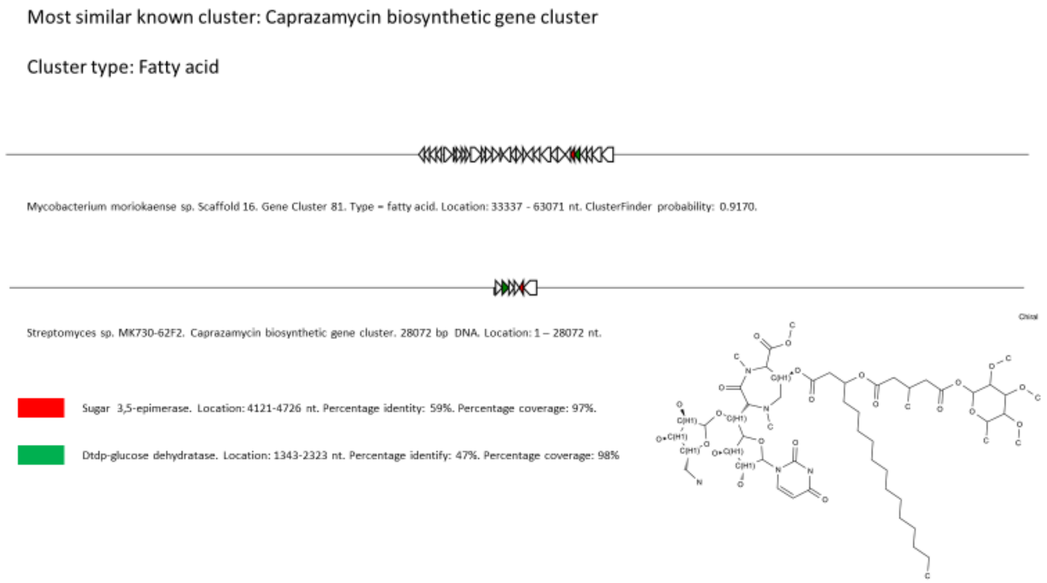

Figure S3. Proposed biosynthetic gene cluster of caprazamycin-like compound in *Mycobacterium* sp.13. The most similar gene cluster from *Streptomyces* sp. MK730-62F2 is shown, with related genes drawn in the same color to highlight inter-cluster rearrangements. The detailed structure of the predicted compound is also displayed.

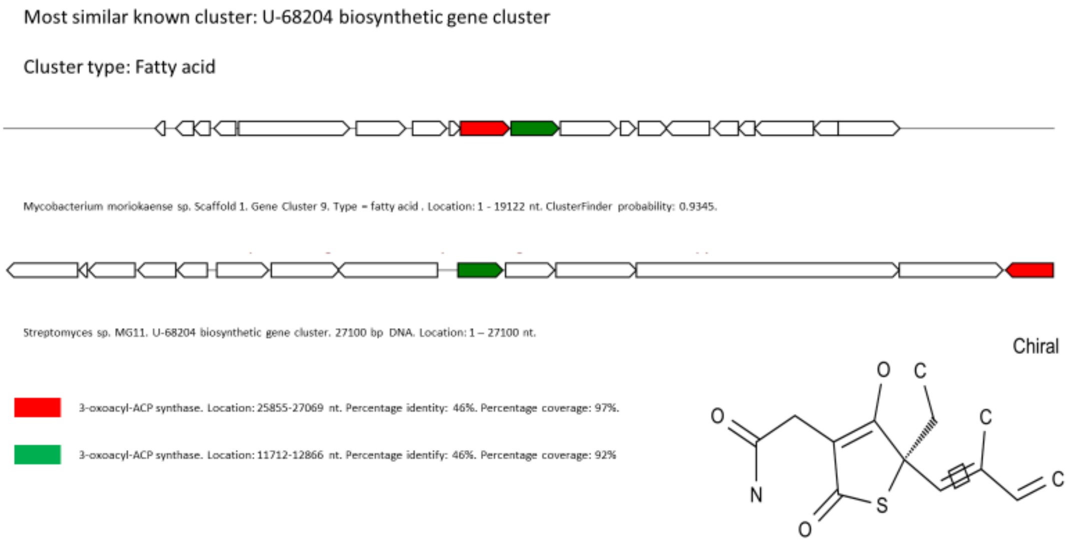

Figure S4. Proposed biosynthetic gene cluster of u-68204-like compound in *Mycobacterium* sp.13. The most similar gene cluster from *Streptomyces* sp. MG11 is shown, with related genes drawn in the same color to highlight inter-cluster rearrangements. The detailed structure of the predicted compound is also displayed.

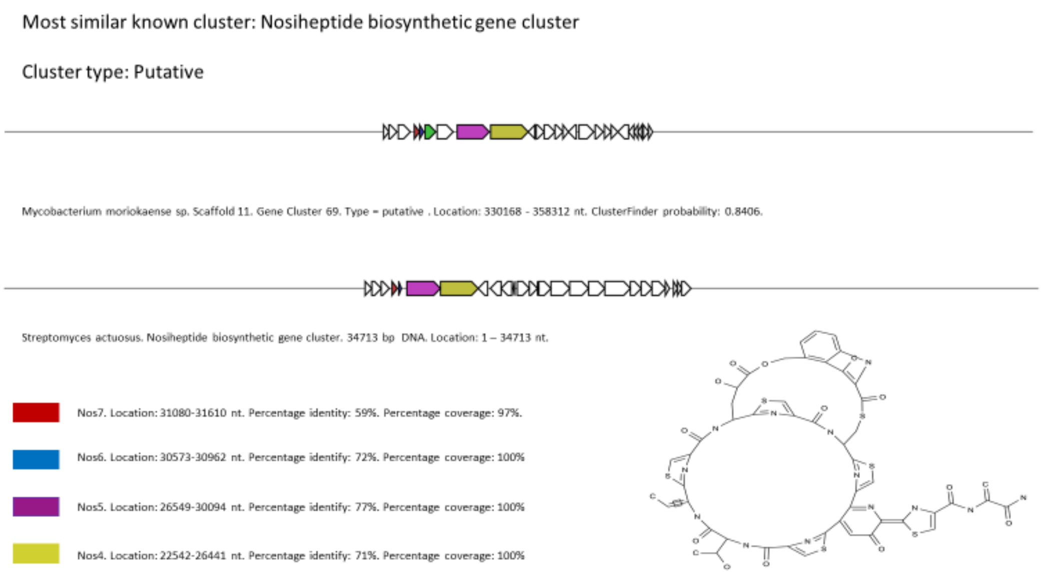

Figure S5. Proposed biosynthetic gene cluster of nosiheptode-like compound in *Mycobacterium* sp.13. The most similar gene cluster from *Streptomyces actuosus* is shown, with related genes drawn in the same color to highlight inter-cluster rearrangements. The detailed structure of the predicted compound is also displayed.

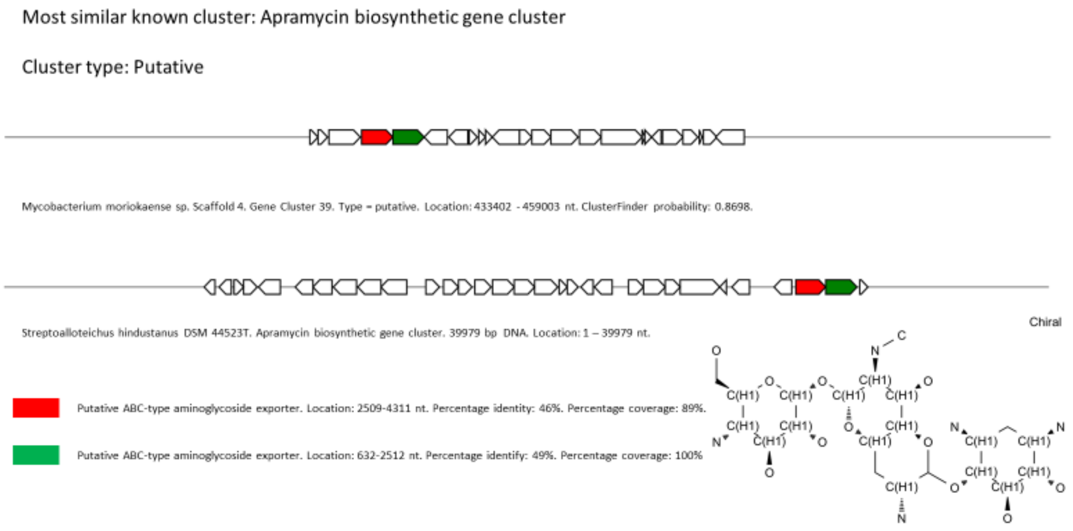

Figure S6. Proposed biosynthetic gene cluster of apramycin-like compound in *Mycobacterium* sp.13. The most similar gene cluster from *Streptoalloteichus hindustanus* DSM 44523^{T} is shown, with related genes drawn in the same color to highlight inter-cluster rearrangements. The detailed structure of the predicted compound is also displayed.

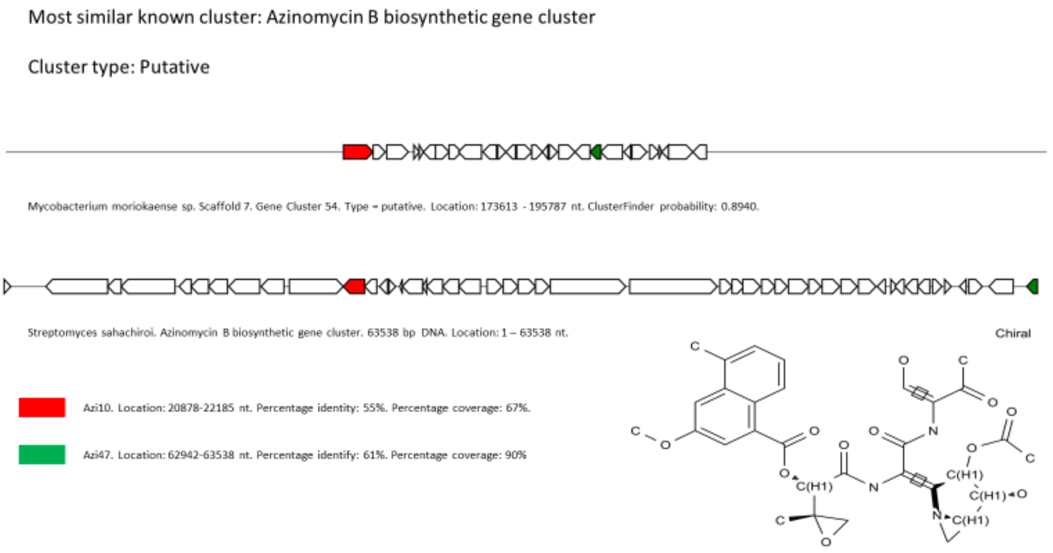

Figure S7. Proposed biosynthetic gene cluster of azinomycin B-like compound in *Mycobacterium* sp.13. The most similar gene cluster from *Streptomyces sahachiroi* is shown, with related genes drawn in the same color to highlight inter-cluster rearrangements. The detailed structure of the predicted compound is also displayed.
